# The Commonplace Book

**Published:** 1918-09-28

**Authors:** 


### RELAXATIONS AND HOBBIES.

### The Commonplace Book.

I HAVE always been interested in this column since I read an article, which appeared some years ago, entitled "Scorpion Hunting on the Karoo." It sounded so delightfully remote, so improbable, so adventurous, that it remains among such mysteries as speaking to the dead, upon the art of which a "practical handbook" has been lately advertised, which I shall never be persuaded to explore. Since then, at too uncertain intervals, I have read of sea voyages, of the life of birds, of a hundred-and-one delights of leisure, but never of my own, a Commonplace Book. With the Victorian era the venerable album seems to have fallen into disuse, and to have been replaced by a vulgar volume containing newspaper cuttings of the odd, the earnest, or the dull, and I find few with Hamlet's habit of jotting down the ripe plums which fall unconsciously from the lips of the casual talker in the train, the street, or the smoking-room. Yet these are often the very jewels of speech, and I have always been "a snapper-up of unconsidered trifles." Since the latest article of the series concerned the collection of antiques, that is to say, of considered things, my own collection must justify itself by a wholly different virtue. Each entry should show spontaneity rather than age, but the reader is warned that the collection is composed of utterances which no one has prized particularly except myself. Since, however, that is presumably the virtue of a hobby, which is essentially peculiar to its amateur, I offer a selection rather to those who are prepared to be bored than to those who hope for amusement.

The book began with quotations only. I could not but get by heart a phrase, a sentence, or a period which once had moved me. By setting it down it was always singing in my ears. Gradually, however, *obiter dicta* were included, such as the racy oath of a tramp, the technical idiom of a trade, the criticism of a man in the train, or the excuse of a shopman who was trying to pass a bad bargain. In dipping casually into the volume I find recorded someone's definition of marriage as "penal servitude for life," and near by an uncle's letter to his nephew, Arthur, in the following terms: "Arthur, now that Autumn's come, eat your fig, enjoy your plum, with currants red and currants white, satisfy your appetite. Spare no apple, spare no peach, spare no nectarine in reach, but yet in moderation so that nought amiss inside may go. Two rules I give: first do not cram too much upon your diaphragm. Next, don't mix gooseberry with grape; 'twill twist you wholly out of shape. Grape with grape is very good: the gooseberry is wholesome food; but grape mixt up with gooseberry, that is just what's bound to lay you flat, to bring the doctor and, what's worse, blue pill, the night commode and nurse." Next to this is the confession of a young man who, having started his career with the maxim "If I cannot be rich, at least let me be luxurious," was asked by an aunt to what his travelling companionship would ultimately lead. He replied, "To suicide," but he has not yet attained his ambition. This is followed by the text of a telegram in which a young fellow who had clandestinely left home for a trip abroad announced his return to his fond and anxious parents. "Prepare the fatted calf," was the whole of his message, and since his home was in a remote and rural place, the telegram soon ran round all that part of the county. A recent addition concerned a conversation in the train with reference to a certain law-suit. "It is dreadful to think," began my *vis-à-vis*, "that there are 47,000 people who are selling their country to conceal their own crimes." "Surely you don't believe that nonsense," retorted the old gentleman, his neighbour. "Indeed I do. I am no party man and follow no one journal, and the thing is said in *all* the newspapers." It was a good example of the state of newspaper hypnosis in which most people go down in confusion to their graves.

Another batch concerns opinions upon books. The first is Gladstone's sublime criticism of some new volume, which he described gravely to be "An able, though concise, book," and succeeded in touching the extreme of fatuity in the phrase. Another was the dictum uttered by a scholar to whom the Tercentenary volumes on Shakespeare were sent by a friend with a request for an opinion on their merits. "Being intended for the gross public this is a highly moral work," he replied, and refused to add anything further on the subject.

Another entry concerns a medical libel action which was related to the writer by a medical man to beguile the tedium of his visit. A certain litigious lady, he said, accused her doctor of an attempt to poison her, and sent the accusation by her solicitors to the doctor through the post. He contented himself with a brief note in which he told the solicitors that their client must be "a bit touched." The solicitors showed this letter to the lady and advised her in her own interests (or was it in theirs?) to take an action for libel. They pointed out that the word "touched" was doubly libellous from the fact that it was an ambiguous expression of which the Court would probably take a grave view, and that it was likely that the Court would rule that the expression meant "suffering from syphilis," since the word "touched" was synonymous with the word "contaminated." The lady took their advice and lost her case, but the solicitors received their fees. *Quod erat faciendum?*

Elsewhere occurs the remark of a man of science, whose amusement it was to tilt at modern ideas. He used to say that the world was indeed ruled by ideas, as Shelley had remarked in his essay on poetry, and that the ideas of any particular generation were those which had slipped into currency through the

**Relaxations and Hobbies**—(*continued*).

style of the poets and thinkers of fifty years or so before. He used to say that present-day fashions, belief in feminism, in equality, in democracy, were merely the legacy of early nineteenth-century writers, and that the newspaper notions of the hour were sprung from the brains of Shelley and John Stuart Mill, and at last had reached those last fastnesses of ignorance, the season-ticket holders. He was particularly severe upon what he called the cant of progress, and his dictum on the subject ran, "The very people who affirm the existence of progress maintain the immutability of morals with the same infatuation as that with which their grandparents maintained the immutability of species."

These scattered remarks from the Commonplace Book will probably deter anyone else from attempting to keep one, and no one, except a Boswell, will refuse to admit that the things which he has heard and omitted to record are finer than the few which he has not been too careless to copy. But a series even of dull passages like the above reminds us how much a remark owes to the moment at which it appears, and how its colour is apt to vanish when the speaker, the scene, and the occasion are no longer there to provide it with its original setting.

